# Timing of Re‐Evaluation After Periodontal Therapy: A Randomized Clinical Trial

**DOI:** 10.1111/jre.70086

**Published:** 2026-02-10

**Authors:** Walter Castelluzzo, Cosimo Rupe, Michele Nieri, Maria di Martino, Marco Zini, Anna Sartori, Alessia Tasco, Folco Spoleti, Luigi Barbato, Francesco Cairo

**Affiliations:** ^1^ Department of Experimental and Clinical Medicine, Research Unit in Periodontology and Periodontal Medicine University of Florence Florence Italy

**Keywords:** nonsurgical periodontal therapy, oral health related quality of life, periodontal re‐evaluation, pocket closure, randomized clinical trial, subgingival instrumentation

## Abstract

**Aim:**

To evaluate clinical and patient reported outcomes after subgingival instrumentation at two different re‐evaluation timings in stage III/IV periodontitis and the influence of clinical and radiographic variables on final outcomes.

**Methods:**

Forty participants were assigned to 3‐month (control) or 6‐month (test) re‐evaluation after steps 1–2 of therapy. The primary outcome was the number of teeth reaching the Endpoints of Treatment (EoT). EoT was defined as a site with PPD < 6 mm or PPD = 4/5 mm without BoP. Secondary outcomes included changes in clinical parameters and in oral health‐related quality of life (OHIP‐14) scores. ANOVA, ANCOVA, mixed‐effects models and multilevel models were applied.

**Results:**

Thirty‐six patients completed the study. Patients allocated to the 3‐month group had 15.8 ± 4.0 teeth reaching the EoT, accounting for 65.8% ± 14.3 of the teeth, while patients assigned to the 6‐month group had 15.5 ± 5.9 (64.9% ± 21.5), without statistically significant differences. Percentages of sites achieving EoT were 68.5% ± 11.6 in the 3‐month group and 71.9% ± 14.4 in the 6‐month group, without statistically significant differences. Final OHIP‐14 scores were 6.5 ± 8.9 in the 3‐month group and 7.3 ± 7.5 in the 6‐month group, without statistically significant differences. Risk for residual pockets at re‐evaluation was influenced by higher baseline PPD (*p* < 0.0001), plaque (PI) at site level (*p* = 0.011), molar tooth (*p* = 0.012), furcation involvement (*p* < 0.0001), shallow intrabony defect (*p* = 0.018), deep intrabony defect (*p* = 0.002).

**Conclusion:**

No difference in clinical and patient‐centered outcomes was observed between groups. NSPT frequently failed to achieve EoT at pockets with intrabony defects, while EoT were frequently achieved at sites with mainly horizontal bony defects.

**Trial Registration:**

ClinicalTrials.gov identifier: NCT06086821 (https://clinicaltrials.gov/study/NCT06086821?cond=re‐evaluation%20periodontal&rank=1)

## Summary

**Background:**

Periodontal re‐evaluation after nonsurgical periodontal therapy (NSPT) is essential to evaluate treatment response, but the optimal timing remains unclear. Healing processes may continue beyond 3 months, but evidence on whether later re‐evaluation improves outcomes is limited.

**Added Value of This Study:**

This randomized controlled trial compared 3‐ and 6‐month re‐evaluation in stage III–IV periodontitis patients. The study found no significant differences between groups in clinical outcomes or patient‐reported quality of life. It strengthens current evidence suggesting that most soft tissue healing is complete within 3 months. The following clinical parameters significantly influenced the outcomes of NSPT: presence of an intrabony defect, site‐level presence of plaque at the re‐evaluation visit, molar teeth, and furcation involvement.

**Clinical Implications:**

Clinicians may perform periodontal re‐evaluation at 3 months without compromising clinical or patient‐reported outcomes. This may allow for earlier identification of residual pockets and may optimize decision‐making for further treatment. Furthermore, the intrabony defect should be considered one relevant prognostic factor influencing the achievement of clinical endpoints after NSPT.

## Graphical abstract

Re‐evaluation at 3 or 6 months resulted in similar clinical and patient‐reported outcomes. NSPT frequently failed to achieve EoT at pockets with deep intrabony defects (≥ 3 mm), while EoT were frequently achieved at sites with mainly horizontal bony defects.

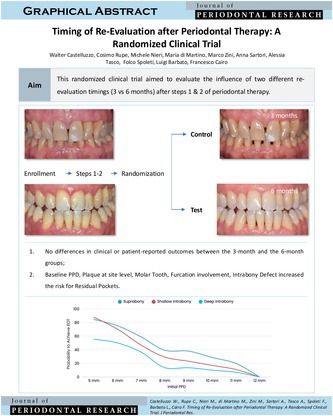

## 1 Introduction

Treatment of periodontitis is based on an incremental stepwise approach, with the first two steps focusing on patients' behavioral changes and supra‐ and sub‐gingival biofilm control [1]. After these initial two phases, in complex cases such as stage III or IV periodontitis patients, the endpoints of therapy (EoT), defined as the absence of sites probing more than 5 mm and not probing more than 3 mm with bleeding on probing [1], may not be completely achieved [2, 3]. In fact, deeper pockets may result in a greater reduction after instrumentation [4], but pocket closure may be less predictable [5].

Periodontal re‐evaluation is performed after the second step to assess reductions in gingival inflammation and pocket depth, gains in clinical attachment, and changes in prognosis. This assessment is essential for evaluating treatment response and determining further clinical needs. In fact, residual pockets (RPs) and furcation involvements have been associated with a higher risk of disease progression and tooth loss during supportive periodontal therapy [6, 7, 8].

Various timings for re‐evaluation after nonsurgical periodontal therapy (NSPT) have been reported, ranging from 2 weeks to 6 months [9]. Classic clinical studies suggested that main soft tissues modifications occur within 3 months after root debridement, but further improvements can be expected up to 6 months [10]. Furthermore, histological studies showed that, while the re‐establishment of junctional epithelium takes place approximately 2 weeks after instrumentation [11] precisely oriented collagen fibers are detected after 2 months [12].

A very recent systematic review [13] showed that the greater clinical improvements are detectable in the first 2 months, although some further changes may occur up to 6 months. Assessing the appropriate timing for re‐evaluation may help clinicians to optimize treatment plan. However, evidence from studies on how different re‐evaluation timings influence clinical outcomes after subgingival instrumentation remains limited [9].

EoT may be reached by means of the first two steps of treatment in the majority of sites [2]. However, several patient‐, tooth‐, and site‐level factors can influence the outcomes of NSPT. Among these are smoking habit, presence of plaque, molar tooth [5], tooth mobility [14], and furcation involvement [15]. Also, bone defect morphology may play a role in the healing process. Pockets associated with intrabony defects have been traditionally considered less responsive to NSPT, even if minimal invasive approaches seem to show promising benefits [16, 17].

Nevertheless, the evidence about the healing of periodontal pocket associated with intrabony defects after NSPT is still controversial, and a recent systematic review highlighted the need for clinical studies addressing this clinical issue [18].

Within this context, the primary aim of this study was to compare the influence of two different timings (3 and 6 months) for periodontal re‐evaluation on clinical and patient‐reported outcomes after NSPT. The secondary aim was to evaluate the possible influence of clinical and radiographic variables on the achievement of EoT following NSPT.

## 2 Methods

### 2.1 Study Design

This study was designed and reported according to the CONSORT statement (http://www.consort‐statement.org) as a superiority, parallel, single‐center RCT with a 1:1 allocation ratio. The study was approved by the local ethics committee (CEAVC 24396) and registered on clinicaltrials.gov (NCT06086821).

Informed consent was obtained from all patients. The study was conducted according to the principles outlined in the Declaration of Helsinki on experimentation involving human subjects.

Two different timings for periodontal re‐evaluation were compared: 3 months (control group) and 6 months (test group). The null hypothesis of the study was that there is no difference between groups in the number of teeth achieving EoT (primary outcome variable).

### 2.2 Facilities, Examiners, and Operators

All diagnostic and therapeutic procedures were conducted at the Department of Experimental and Clinical Medicine, Research Unit in Periodontology and Periodontal Medicine, University of Florence, Florence, Italy. A single examiner (C.R.) was trained for clinical measurement and attended a calibration session, reporting an intraclass correlation coefficient of 0.90 (95% CI 0.85; 0.93) for probing depth measurements, and a Cohen's *k* score of 0.93 (95% CI 0.88; 0.98) for EoT. Periodontal therapies were administered by 2nd, or 3rd‐year students enrolled in the EFP‐accredited post‐graduate program in Periodontology and Implant Dentistry at the University of Florence. Before experimental procedures, the selected operators previously treated at least 30 patients/each with NSPT. All treatment delivery was performed under tutor supervision.

### 2.3 Eligibility and Informed Consent

Patients meeting the eligibility criteria for the study were identified among those referred to or seeking care at the Department of Periodontology at AOU Careggi in Florence.

*Inclusion criteria:*

- Adults (≥ 18 years old) with a diagnosis of stage III or stage IV periodontitis;
- At least 20 teeth present.

*Exclusion criteria:*

- Patients receiving periodontal therapies in the past 12 months;
- Patients receiving antibiotics in the past 3 months;
- Known medical conditions or drug therapies that affects clinical signs or the progression of periodontitis or that affect the outcomes of periodontal therapy;
- Pregnancy;
- Compromised medical conditions requiring prophylactic antibiotic prescription.

The study staff verified eligibility, explained the study, answered any research‐related questions, and obtained signed informed consent before initiating any research procedure.

### 2.4 Steps 1 and 2 of Periodontal Therapy

After the baseline examination, patients were informed on periodontitis and were provided with detailed and individual instructions on self‐performed oral hygiene procedures (OHP). Additionally, patients were advised to limit and possibly cease lifestyle habits contributing to the progression of periodontitis. Then, a careful subgingival instrumentation was administered according to a fullmouth protocol using periodontal tips on ultrasonic devices and mini Gracey's curettes under 3–4× magnification. No additional therapies were used. The mean length of the procedure was then calculated.

Monthly, patients were visited, and OHP were renewed and corrected, if necessary. In particular, the full mouth plaque score (FMPS) was assessed by the operators, and if the value exceeded 25%, oral hygiene instructions were repeated. Patients were asked to demonstrate how they performed OHP at home, and the operators checked and corrected, in case, these maneuvers. In addition, the size of the interproximal brushes was checked and adjusted, if necessary, according to anatomy of the interdental spaces. This approach ensured that the re‐evaluation could estimate the effect of a single subgingival instrumentation procedure.

### 2.5 Allocation Concealment and Randomization Process

Randomization was performed using a computer‐generated list by a blinded operator and allocation concealment was achieved by opaque, continuously numbered sealed envelopes.

After the delivery of periodontal therapy, an investigator not involved in the clinical procedures (L.B.) opened the sealed envelope. If the patient was assigned to the Control group (3 months), two oral hygiene maintenance appointments were scheduled (at 1 and 2 months), along with a 3‐months (±7 days) appointment for re‐evaluation. Conversely, if the patient was assigned to the Test group (6 months), a series of five, monthly appointments were scheduled, followed by a final appointment at 6 months (±7 days) for periodontal re‐evaluation. The examiner was unaware of the allocation and did not perform any of the monthly visits.

### 2.6 Variables

At baseline and at re‐evaluation the following clinical parameters were evaluated using a periodontal probe (PCP‐UNC 15 probe; Hu‐Friedy Group, Chicago, IL, USA):

- PPD (Probing pocket depth): distance in mm between the gingival margin (GM) and the base of the probable pocket:
- Rec (Gingival recession): distance in mm between the cement enamel junction (CEJ) and the GM (this value was positive if the CEJ was coronal to the GM and negative if the CEJ was apical to the GM); in the presence of prosthetic full crowns, the prosthetic margin was used as the reference point;
- BoP (Bleeding on Probing): scored dichotomously within 15 s after probing;
- PI (Plaque Index): scored dichotomously;
- Tooth mobility: scored as class 0, 1, 2, or 3 [19];
- CAL (clinical attachment level): estimated as the sum of PD and REC;
- Horizontal furcation involvement was evaluated by means of a Nabers probe (2 N probe; Hu‐Friedy Group, Chicago, IL, USA) and scored [20];
- FMPS (full mouth plaque score) and FMBS (full mouth bleeding score): expressed as the percentage of sites with detectable plaque upon probe passage and the percentage of bleeding sites upon probing;
- EoT were dichotomously scored at re‐evaluation at all sites, coupling data on PPD and BoP. According to the EFP clinical guidelines [1], EoT was defined as a site with PPD < 6 mm or PPD = 4/5 mm without BoP.
- At re‐evaluation, the number of teeth reaching EoT (primary outcome) was calculated.
- Furthermore, the number of teeth with deep (i.e., showing at least one site with PPD ≥ 6 mm) and moderate residual pockets (i.e., PPD = 4/5 mm with BoP, but not deeper probing) was calculated.

Full‐mouth radiographs using the parallel cone technique were also collected at baseline.

Perceptions of oral health‐related quality of life, using the Italian version of the OHIP‐14 (oral health impact profile) questionnaire, were assessed at baseline and at re‐evaluation [21]. The questionnaire was previously validated and showed a Cronbach's alpha of 0.90 [22].

### 2.7 Radiographic Examination

All radiographs of selected sites were analyzed using a specific software (Gendex VixWin, KaVo Dental, Biberach, Germany). The following radiographic landmarks [23, 24, 25] were identified:

- Cementoenamel junction (CEJ);
- Root apex (RA);
- Bone crest as the most coronal portion of the intrabony defect (BC);
- Defect bottom as the most apical portion of the intrabony defect (BD).

In multirooted teeth, root associated with the defect was selected. For maxillary teeth, buccal roots were always used. To obtain the necessary linear measurements, perpendicular lines were projected from CEJ, BC, and BD to the long axis of the tooth, on points CEJ1, BC1, and BD1 [24]. The distances between CEJ1 and BC1 and BC1 and BD1 were then calculated.

**FIGURE 1:**
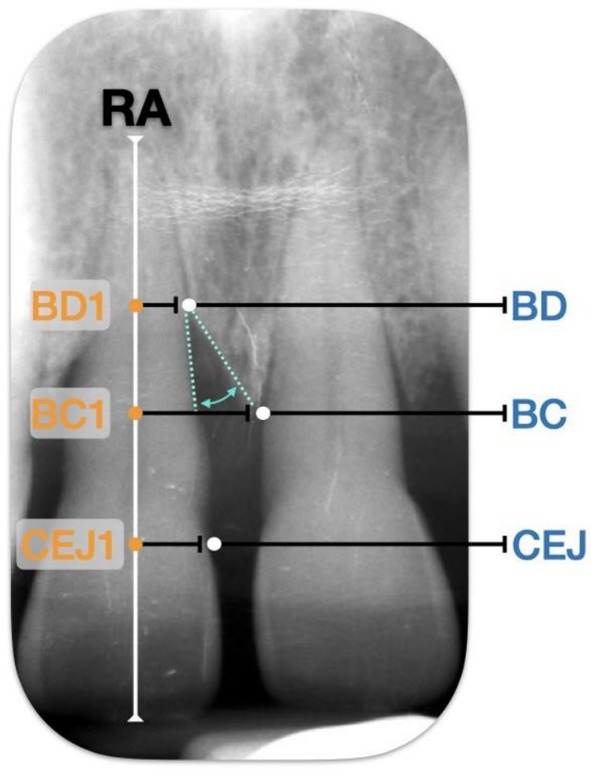
Example of the landmarks used for radiographic measurements. BC, bone crest (most coronal portion of the intrabony defect); BC1, projection of BC on the long axis of the tooth; BD, defect bottom (most apical portion of the intrabony defect); BD1, projection of BD on the long axis of the tooth; CEJ, cementoenamel junction; CEJ1, projection of CEJ on the long axis of the tooth; RA, root apex.

The following variables were then recorded, as shown in Figure 1:

- CEJ1‐RA: the distance between the CEJ and RA, that is, root length. This linear distance was used to identify the long axis of the tooth;
- CEJ1‐BC1: the length, in mm, of the horizontal bone loss;
- BC1‐BD1: the depth, in mm, of the intrabony defect;
- Radiographic angle: The defect angle was defined by two lines. The first line was constructed along the root surface from RA to CEJ1. The second line followed the defect surface from BD to BC, the most coronal part of the defect where it touched the neighboring tooth.

Intrabony defects were further clustered as follows:considering the radiographic depth:

- Deep: when the intrabony component was ≥ 3 mm;
- Shallow: when the intrabony component was < 3 mm.

The radiographic angle of the intrabony defect was categorized as wide (≥ 37°) or narrow (< 37°) [26, 27].

### 2.8 Training for Defect Morphology Estimation

All radiographic analyses were performed by a previously trained single examiner (M.Z.), unaware of the clinical procedures. A calibration exercise was carried out for the radiographic assessments, including 20 duplicate measurements of bone levels and defect angles performed on separate days on x‐rays different from those used in this study. The intra‐class correlation coefficients were between 0.86 and 0.82 for linear and angle measurements, indicating good reproducibility in radiographic measurements.

### 2.9 Data Entry and Analysis

All anonymized data were entered in a dedicated database. Access was restricted to personnel responsible for data entry and study investigators. Subsequently, the data underwent a thorough proofreading process to identify and correct any entry errors.

### 2.10 Sample Size

Considering that no data were available in the literature on the mean number of teeth reaching EoT and the corresponding standard deviation when comparing different re‐evaluation timings, the sample size calculation was based on a standardized effect size. Specifically, the study was powered to detect a between‐group difference corresponding to one standard deviation in the number of teeth reaching EoT. With a two‐sided significance level of 5% and a power of 80%, 17 patients per group were required. To account for an anticipated dropout rate of 15%, the final sample size was increased to 20 patients per group, for a total of 40 patients.

### 2.11 Statistical Analysis

Descriptive analyses were performed using means and standard deviations for quantitative variables and frequencies and percentages for qualitative variables. ANOVA tests were conducted for the number and percentages of teeth reaching the EoT (primary outcome) at the patient level. A further explorative analysis was conducted to detect differences between groups in terms of number of teeth presenting with moderate RPs (4–5 mm) or at least one deep RP (≥ 6 mm). ANCOVA tests were used to analyze changes between baseline and re‐evaluation examinations for FMPS, FMBS, PPD, Rec, CAL, and OHIP‐14 values, with baseline values as covariates. Different subgroup analyses were performed, respectively including sites with a baseline PPD ≥ 6 mm, deep intrabony defect and FI.

A modified intention‐to‐treat analysis was performed, excluding drop‐outs [28].

Considering that this primary statistical analysis did not highlight differences between groups, the data from both groups were pooled to perform a secondary explorative analysis aimed at evaluating the predictors of RPs. To specifically investigate the impact of supra‐ and intra‐bony defects on treatment outcomes, only interproximal sites that, at baseline, exhibited PPD ≥ 6 mm or PPD ≥ 4 mm with BoP were included in the analysis. A multilevel logistic regression model with three levels of variability (patient, tooth, and site) was built to analyze RPs (dichotomous outcome). The proportion of variance explained at site, tooth and patient level was calculated as their relative contribution to the final outcomes. Random effects were defined a priori and were assumed to be uncorrelated at different levels.

Bivariate analyses were conducted, considering every single variable as a predictor variable. The following variables were included in the bivariate analysis: sex, age, smoking, stage of periodontitis, grade of periodontitis, FMBS, FMPS (patient level), mobility, tooth type (tooth level), PPD, BoP, PI, FI, presence of a deep intrabony defect, presence of a shallow intrabony defect, intrabony defect depth, intrabony defect angle (site level). A multilevel, stepwise backward analysis was performed using the significant variables in the bivariate analyses. The level of significance (*p* > 0.05) was considered as exclusion criterion for the stepwise backward analysis. Interaction terms were included if statistically significant. This analysis was defined a priori. Model fit was assessed using the Akaike information criterion (AIC) to compare the full model to an initial null model. To examine potential autocorrelation in the model residuals, the Durbin–Watson statistic was applied. Potential multicollinearity was assessed calculating Variance Inflation Factors (VIF) for all predictors.

A descriptive graph was realized describing the probability of achieving EoT for different intrabony defect depths and baseline PPDs. A residual analysis was performed for all models used in the study to verify the statistical assumptions of the models themselves.

Statistical analyses were formed with the following softwires: IBM SPSS Statistics software version 25 (IBM Corp, Armonk, NY, USA) and JMP version 19 (SAS Institute Inc.).

## 3 Results

### 3.1 Study Population

**FIGURE 2:**
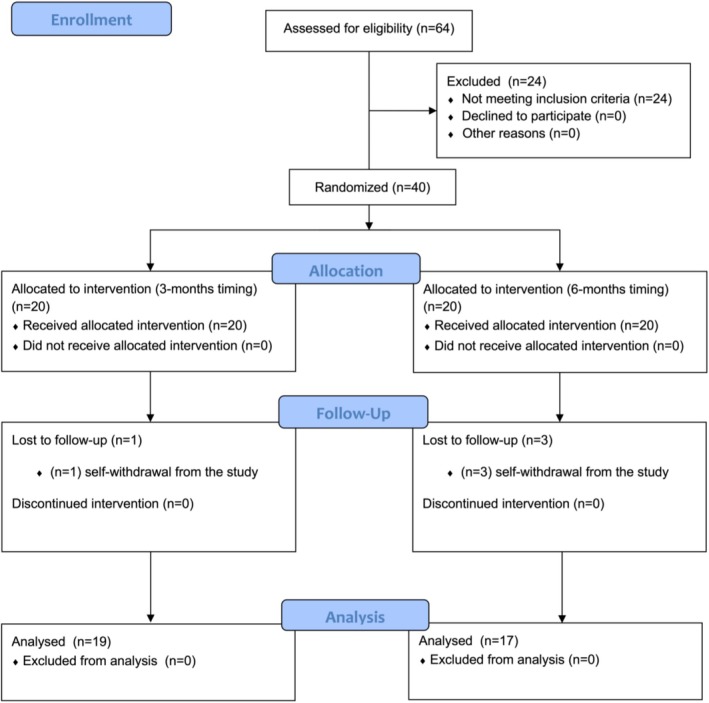
CONSORT diagram of the study.

The flow diagram of the study is presented in Figure 2. An original cohort of 64 patients was screened for eligibility. Forty patients were included and randomized. Four patients were lost to follow‐up, three in the 6‐months group and one in the 3‐months group. A total of 36 patients, accounting for 946 teeth and 5676 sites, completed the study. The mean length of the procedure was 143.2 min (SD: 37.1) in the 6‐months group and 139.5 min (SD: 33.4) in the control group, without significant differences.

Baseline demographic and clinical characteristics are summarized in Table 1.

**TABLE 1:** Baseline demographic and clinical characteristics.

Variable	Control group (3 months) (*N* = 20)	Test group (6 months) (*N* = 20)
Sex (Female)	9 (45%)	9 (45%)
Age (years)	57.3 (10.5)	55.5 (8.2)
Stage (4)	7 (35%)	8 (40%)
Grade (C)	12 (60%)	16 (80%)
Generalized	20 (100%)	19 (95%)
Smokers	9 (45%)	7 (35%)
< 10 cigarettes/day	2 (10%)	5 (25%)
≥ 10 cigarettes/day	7 (35%)	2 (10%)
Teeth (*n*)	23.7 (3.0)	23.6 (3.2)
Teeth with PPD 4–5 mm and BoP+ or PPD ≥ 6 mm (Baseline, n)	17.7 (3.9)	19.2 (4.0)
Teeth with moderate PPD (4–5 mm) BoP+ (Baseline, *n*)	6.7 (3.9)	7.4 (4.3)
Teeth with deep PPD (≥ 6 mm) (Baseline, *n*)	11.1 (5.8)	11.8 (5.8)
FMPS (%)	68.0 (15.0)	76.6 (17.5)
FMBS (%)	56.6 (16.8)	66.1 (17.2)
PPD ≤ 3 mm (*n*)	82.1 (23.3)	72.4 (26.4)
PPD 4–5 mm (*n*)	34.8 (12.0)	39.3 (11.9)
PPD ≥ 6 mm (*n*)	24.0 (16.0)	29.8 (20.5)
Mean PPD (mm)	3.8 (0.6)	4.0 (0.7)
Mean Rec (mm)	0.8 (0.9)	0.8 (1.0)
Mean CAL (mm)	4.6 (1.3)	4.8 (1.3)
OHIP‐14	15.8 (9.3)	15.7 (9.3)
Intrabony Defects (*n*)	8.3 (7.7)	8.3 (6.9)
Sites with Furcation Involvement (*n*)	13.1 (6.1)	15.1 (7.1)
Bony defects (pooled data)		
Suprabony defects (*n* and %)	1207 (78.4%)	
Shallow intrabony defect (< 3 mm) (*n* and %)	226 (14.7%)	
Deep intrabony defect (≥ 3 mm) (*n* and %)	107 (6.9%)	
Intrabony defect depth (mean; mm)	3.65 (2.09)	

Abbreviations: CAL, clinical attachment level; FMBS, full mouth bleeding score; FMPS, full mouth plaque score; OHIP, oral health impact profile; PPD, probing pocket depth; Rec, recession.

### 3.2 Clinical Outcomes

At re‐evaluation, patients allocated in the 3‐month group had 15.8 ± 4.0 teeth reaching the EoT, while patients assigned at the 6‐month group had 15.5 ± 5.9 (difference: −0.3; 95% CI [−3.6 to 3.1]; *p* = 0.876). Additionally, there was no statistically significant difference in the number of teeth with moderate RPs (4–5 mm) (difference: 0.5; 95% CI [−1.2 to 2.1]; *p* = 0.563) or those presenting with at least one deep RP (≥ 6 mm) (difference: −0.4; 95% CI [−2.2 to 1.5]; *p* = 0.708) between the two groups.

Percentages of sites achieving EoT was 68.5% ± 11.6% in 3‐month group and 71.9% ± 14.4% in 6‐month group (difference: 3.5; 95% CI [−5.4 to 12.3]; *p* = 0.430). For details see Table 2.

**TABLE 2:** Clinical outcomes (ANOVA): differences between groups for primary and secondary outcomes at re‐evaluation after NSPT.

Variable	Control group (3 months) (*N* = 19)	Test group (6 months) (*N* = 17)	Difference	95% CI	*p*
Teeth reaching EoT (*n*)	15.8 (4.0)	15.5 (5.9)	−0.3	−3.6; 3.1	0.876
Teeth reaching EoT (%)	65.8 (14.3)	64.9 (21.5)	−1.2	−13.5; 11.0	0.840
Teeth with moderate RPs (Final, *n*)	3.5 (1.7)	3.9 (3.0)	0.5	−1.2; 2.1	0.563
Difference with baseline (Final, *n*)	3.4 (4.2)	3.9 (5.8)	0.5	−2.9; 3.9	0.785
Teeth with deep RPs (*n*)	4.1 (3.1)	3.7 (2.3)	−0.4	−2.2; 1.5	0.708
Difference with baseline (Final, *n*)	6.7 (3.2)	7.3 (3.8)	0.6	−1.7; 3.0	0.602
Total EoT (*n*)	125.6 (18.5)	124.6 (20.8)	−1.1	−14.3; 12.2	0.873
Total EoT (%)	87.3 (6.2)	87.2 (8.6)	−0.0	−5.2; 5.1	0.989
EoT (*n*)	31.9 (13.0)	40.2 (14.9)	8.2	−1.2: 17.7	0.085
EoT (%)	68.5 (11.6)	71.9 (14.4)	3.5	−5.4; 12.3	0.430
Sites with baseline PPD ≥ 6 mm					
EoT (%)	49.8 (17.9)	53.7 (18.0)	3.9	−8.3; 16.1	0.522
Sites with deep intrabony defects (≥ 3 mm)					
EoT (%)	28.1 (45.3)	44 (50.1)	15.9	−2.5; 34.3	0.107
Sites with FI					
EoT (%)	45.2 (50.1)	53.8 (50.1)	8.6	−4.7; 21.9	0.231

*Note:* Total EoT = sites that reached the EoT on the total of included sites (i.e., also sited with baseline pockets < 4 mm); EoT = sites that reached the EoT on the total of sites that, at baseline, had PPD ≥ 6 mm or PPD ≥ 4 mm with BoP; moderate RPs: 4–5 mm with BoP; deep RPs: ≥ 6 mm.

Abbreviations: EoT, endpoint of therapy; PPD, probing pocket depth; RPs, residual pockets.

NSPT resulted in a reduction in both the FMPS and FMBS, as well as a decrease in the mean PPD, increase of mean REC and decrease in the number of pockets, with no statistically significant differences between the two groups. The final OHIP‐14 score was 6.5 ± 8.9 in 3‐months and 7.3 ± 7.5 in 6‐months group, without statistically significant differences between groups (difference: −0.8; 95% CI [−5.1 to 3.4]; *p* = 0.692). For details see Table 3.

**TABLE 3:** Clinical and patient‐reported outcomes (ANCOVA): secondary outcomes at re‐evaluation after NSPT.

Variable	Control group (3 months) (*N* = 19)	Test group (6 months) (*N* = 17)	Difference	95% CI	*p*
FMPS (%)	16.1 (7.7)	22.0 (12.6)			
FMPS change (%)	52.8 (13.9)	52.3 (22.4)	5.6	−1.6; 12.8	0.123
FMBS (%)	16.9 (9.7)	17.3 (8.9)			
FMBS change (%)	39.2 (15.8)	46.0 (13.3)	1.5	−4.4; 7.4	0.605
PPD (mm)	2.9 (0.4)	2.8 (0.4)			
PPD change (mm)	0.8 (0.3)	1.0 (0.4)	0.14	−0.01; 0.29	0.075
Rec (mm)	1.3 (0.9)	1.6 (0.9)			
Rec change (mm)	0.6 (0.5)	0.8 (0.4)	0.23	−0.05; 0.52	0.106
CAL (mm)	4.1 (1.1)	4.5 (1.1)			
CAL change (mm)	0.2 (0.4)	0.2 (0.5)	−0.08	−0.33; 0.17	0.524
OHIP‐14	6.5 (8.9)	7.3 (7.5)			
OHIP‐14 change	9.3 (7.3)	8.5 (7.7)	−0.8	−5.1; 3.4	0.692
PPD re‐ev (mm)	4.7 (1.7)	4.5 (1.9)			
Only sites with PPD ≥ 6 mm					
PPD change (mm)	2.3 (1.5)	2.6 (1.8)	0.24	−0.13; 0.62	0.201
Rec re‐ev (mm)	1.9 (1.8)	2.3 (1.8)			
Rec change (mm)	1.0 (1.3)	1.2 (1.4)	0.36	−0.09; 0.81	0.110
CAL re‐ev (mm)	6.6 (2.4)	6.8 (2.7)			
CAL change (mm)	1.2 (1.8)	1.4 (2.1)	−0.01	−0.38; 0.36	0.964

Abbreviations: CAL, clinical attachment level; FMBS, full mouth bleeding score; FMPS, full mouth plaque score; OHIP, oral health impact profile; PPD, probing pocket depth; Rec, recession.

PPD reduction did not significantly differ between groups (difference: 0.14; 95% CI [−0.01 to 0.29]; *p* = 0.074). For sites with a baseline PPD ≥ 6 mm, a greater PPD reduction in the 6‐months group (2.6 ± 1.8 mm) compared to 3‐months (2.3 ± 1.5 mm) was found, although this difference was not statistically significant (difference: 0.24; 95% CI [−0.13 to 0.62]; *p* = 0.201). Percentages of sites achieving EoT for these sites was 49.8% ± 17.9% in 3‐months and 53.7% ± 18.0% in 6‐months group (difference: 3.9; 95% CI [−8.3 to 16.1]; *p* = 0.522). For deep intrabony defects, a tendency towards higher percentage of EoT achievement was found in the 6‐months group (difference: 15.9; 95% CI [−2.5 to 34.3]; *p* = 0.107). For details see Tables 2 and 3.

### 3.3 Radiographic and Clinical Predictors for EoT

Since the primary analysis did not reveal any differences in any of the variables under investigation, a secondary analysis was conducted by pooling the data from both groups. A total of 671 teeth and 1540 interdental sites were analyzed. There were 1207 suprabony defects and 333 intrabony defects, the mean depth was 3.65 mm ± 2.09. Among these, 107 were deep (≥ 3 mm) and the mean radiographic depth was 5.46 mm ± 1.82.

**FIGURE 3:**
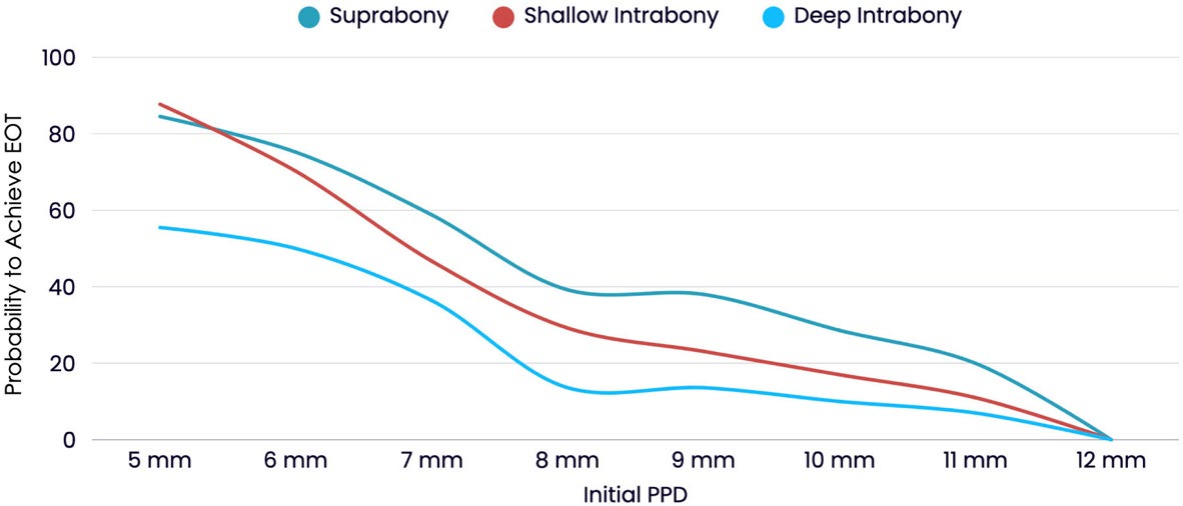
Descriptive graph considering EoT achievement for different radiographic depths of the defects and initial PPD. EoT, endpoint of therapy; PPD, probing pocket depth.

Deep intrabony defects were associated to lower probability of achieving site‐level EoT, irrespective of the initial PPD. For initial PPD ≥ 7 mm, shallow intrabony defect showed lower probability of EoT compared to suprabony ones (Figure 3).

Results of the stepwise backward multilevel logistic regression for RP are showed in Table 4. The following variables significantly increased the probability of RPs: baseline PPD (OR: 1.84; 95% CI [1.67 to 2.02]; *p* < 0.0001), plaque at site level at periodontal re‐evaluation (OR: 1.47; 95% CI [1.09 to 1.99]; *p* = 0.011), molar tooth (OR: 1.61; 95% CI [1.11 to 2.34]; *p* = 0.012), furcation involvement (OR: 2.12; 95% CI [1.39 to 3.22] *p* < 0.0001), shallow intrabony defect (OR: 1.60; 95% CI [1.08 to 2.37] *p* = 0.018; ref. suprabony defect), deep intrabony defect (OR: 2.36; 95% CI [1.37 to 4.07]; *p* = 0.002; ref. suprabony defect).

**TABLE 4:** Multilevel logistic regression: analysis of factors associated with residual pockets (site level).

Variable	Odds ratio	95% CI	*p*
Baseline PPD	1.84	1.67; 2.02	< 0.0001
Plaque (at re‐evaluation)			
No	1.0 (Ref.)		
Yes	1.47	1.09; 1.99	0.011
Molar tooth			
Non molar	1.0 (Ref.)		
Molar	1.61	1.11; 2.34	0.012
Furcation involvement			
No	1.0 (Ref.)		
Yes (any grade)	2.12	1.39; 3.22	< 0.0001
Intrabony defect			
Suprabony defect	1.0 (Ref.)		
Shallow intrabony defect	1.60	1.08; 2.37	0.018
Deep intrabony defect	2.36	1.37; 4.07	0.002

The estimated relative contribution to the overall variance of *‘residual pocket’* outcome was 77.8% for site‐, 14.7% for tooth‐, 7.5% for patient‐related factors (See also Data S1). AIC for the final model was 8478.52, while AIC for the null model was 8530.27.

## 4 Discussion

The primary aim of this study was to compare the efficacy of subgingival instrumentation in stage III/IV periodontitis patients at two different time points: 3 and 6 months after treatment delivery.

Use of varying observation time‐points is not common in periodontal research and may help to assess potential benefits purely related with time after healing [29]. Some authors have suggested that greater PPD reduction and CAL gain may be obtained at longer follow‐ups after NSPT [10, 13].

This study randomly allocated two observation times after the same treatment modality to investigate whether time per se may have a beneficial effect after subgingival instrumentation.

In the present study, no statistically significant difference was observed between groups in the number of teeth achieving the EoT, thus failing to demonstrate additional benefits of extending the observation period from 3 to 6 months. Nevertheless, a slight, but clinically negligible difference was reported, favoring the control group (0.3). Very interestingly, the wide CI (−3.6; 3.1) for the difference between groups suggests that, at least in a subset of the sample, the impact of timing of re‐evaluation may have been higher, warranting further investigations to demonstrate the non‐inferiority between different re‐evaluation timings.

In the two groups, there were approximately 8 teeth per patient on average with RPs at re‐evaluation, about 4 of which had moderate pockets (< 6 mm) and about 4 had deep pockets (≥ 6 mm). This value is very similar to what was reported in a larger study [30], which documented an average of 7.9 teeth with RPs in both study groups among patients with stage III and IV periodontitis.

Furthermore, 68.5% of sites in the 3‐month group and 71.9% in the 6‐month group achieved EoT. These values are very similar to those reported in a recent systematic review on the effectiveness of subgingival instrumentation [2] and strengthen the reliability of the present findings, as the results closely aligns with the findings of the most recent evidence [30].

In this sample of patients, who successfully completed and achieved the therapeutic goals of Steps 1 and 2, approximately 13% of sites will need additional treatment during Step 3. This aligns well with the 11.7% of RPs after NSPT, as reported by a recent systematic review [3]. Both nonsurgical or surgical approaches may be suggested for these patients considering different residual defects [31, 32]. Nevertheless, since an average of four teeth per patient in both groups exhibit at least one deep pocket (≥ 6 mm), it is plausible to hypothesize that most of these patients will require surgical therapy, as suggested by the EFP clinical practice guideline [1].

It should be noted that in this study, contrary to previous investigations on NSPT, the number of teeth reaching EoT was selected as the primary outcome variable, as previously suggested [33]. Thus, while proportion of sites that achieve EoT is relevant in describing treatment performance, is not sufficient to establish the treatment needs for an individual tooth or at patient level. However, it should be underlined that several factors other than PPD and BoP contribute to determine surgical decisions, both at patient (e.g., smoking, FMPS, FMBS), tooth (e.g., molar, furcation involvement, prosthetic needs, mobility), and site level (e.g., bone defect, gingival recession). Consequently, the results of this study should not be interpreted as guidance for the timing of surgical intervention.

The analysis conducted exclusively on sites with a PPD ≥ 6 mm at baseline revealed a not significant difference of 0.24 mm greater PPD reduction favoring the 6‐months group (*p* = 0.201), very similar to the 0.23 mm reported in a recent systematic review [13]. This difference is consistent with the literature [4, 10, 34], which collectively suggest greater PPD reduction after additional 3 months, although the clinical relevance remains unclear. In fact, percentages of sites achieving EoT for these deeper sites did not significantly differ between the two groups: 49.8% in the 3‐months group and 53.7% in the 6‐months group, with similar need for further treatment.

When assessing patient‐reported outcome measures, treatment resulted in a consistent reduction in the OHIP‐14 score. This validated questionnaire [21, 22] is the most widely used method in the literature to assess the impact of periodontitis and improvement of periodontal health on quality of life [35]. In the present sample of patients, the first two steps of periodontal therapy led to an improvement of approximately 9 points in both groups, with no statistically significant differences between them. The results reported in our study are consistent with relevant literature on the topic [36, 37, 38]. An improvement of approximately 6.5 points after 3–4 weeks and about 9 points after 6–12 weeks following the delivery of NSPT was reported [36]. The similarity in results between the two study groups may be well explained by the fact that the clinical outcomes were also very similar, resulting in comparable improvements in periodontal conditions and corresponding responses in the questionnaire.

Since the healing response of pockets with different bone characteristics is not fully understood, the secondary aim of this study was to investigate the potential influence of radiographic and clinical variables on the achievement of therapeutic endpoints following NSPT. Interestingly, both shallow (*p* = 0.018) and deep (*p* = 0.002) intrabony defects resulted to be independent risk factors for RPs compared to suprabony defects. The probability to achieve EoT decreased with each additional millimeter of intrabony defect depth, being lower than 50% for bony defect measuring at least 4 mm. These findings agree with a very recent study [39] showing that suprabony defects were 2.60 times more likely to achieve pocket closure compared with intrabony defects.

The reason behind this clinical response is not completely understood. Healing at intrabony defects may occur due to the formation of a long junctional epithelium and changes in alveolar bone [25]. Nevertheless, it can be hypothesized that complete healing of the intrabony component is unpredictable, probably due to the difficulty of fully removing the subgingival biofilm. It has been suggested that, in the absence of a persistent host inflammatory response, NSPT promotes healing by stabilizing a blood clot over the root and may enhance new bone formation [40]. It is noteworthy that recent RCTs have shown that a nonsurgical approach enabling more efficient subgingival instrumentation was not inferior to papilla preservation flap surgery for the treatment of both shallow [32] and deep residual defects [41].

On the other hand, intrabony defects have been associated with a distinct molecular profile of gingival crevicular fluid compared to periodontally healthy sites, suggesting the presence of senescent cells, which may contribute to slower and less effective healing even after effective subgingival instrumentation [42]. In such cases, minimally invasive surgical procedures appear to be more effective than NSPT [31].

Interestingly, both shallow and deep defects showed a similar percentage of EoT achievement (approximately 35%). As shown in Figure 3, for shallower baseline PPDs, a difference in pocket closure between shallow and deep defects was noticeable, while for deeper pockets, the difference tends to disappear, underscoring the fundamental influence of baseline PPD on clinical outcomes. It can be speculated that baseline PPD, by influencing the likelihood of biofilm removal [43], may mitigate differences between defect depths. However, as PPD increases, the presence of an intrabony defect may represent a negative predictor of treatment, potentially reducing the chances of complete biofilm removal and complicating the healing of the residual pocket.

In this study, baseline PPD (*p* < 0.0001), presence of plaque (*p* = 0.011), molar tooth (*p* = 0.012), and furcation involvement (*p* < 0.0001) all had a significant influence on the probability of RPs at site‐level. These findings confirm the critical impact of these factors, as previously reported [5, 14, 44].

Limits of the study may be the single‐center design or may be related to operator variability since treatment was provided, even if supervised, by EFP postgraduate students. It is important, however, to notice that specific education and calibration processes were conducted before experimental procedures to standardize treatment quality (i.e., treating at least 30 patients each before the beginning of the trial). Conversely, the achieved outcomes seem to support the effectiveness of the delivered therapy since they are similar to those reported in current literature. Furthermore, clinical data were collected in a single session to minimize the risk of alteration due to multiple probing during healing [45]. On the other hand, this approach might have prevented further insight into the temporal dynamics of healing, which would have been possible by repeating the assessments throughout the study.

Also, the study might have been underpowered for the evaluation of the secondary outcomes and for the multilevel analysis. Furthermore, the sample size calculation itself was not based on pre‐existing data, thus potentially limiting the overall power of the study.

Also, the increased number of visits in the 6‐months group may have influenced patient behavior and plaque control, potentially affecting clinical outcomes independently of healing dynamics.

Further maturation of healing and improvement in clinical parameters can be achieved only if patients consistently maintain optimal oral hygiene. Without such strict follow‐up protocol, the 6‐months group would have risked a relapse in oral hygiene behaviors or motivation, rather than the expected improvements. Similarly, the monthly visits could also have influenced patient‐reported outcomes since more clinical contact (5 vs. 2 interim visits) could have itself influenced patient perceptions.

These aspects should be carefully considered when assessing the possible generalizability of the present findings. Another limitation of the study may be the lack of a third allocation group. A periodontal re‐evaluation at 4–6 weeks could have provided additional insights. Further studies might clarify this issue.

As for the secondary analysis, a further limitation should be highlighted: since radiographic examination was bi‐dimensional, it was performed only at interdental sites, meaning that, in case of defect extending at buccal/lingual sites, it was not possible to investigate a possible influence on the healing pattern, thus limiting the generalizability of these findings to the whole dentition, especially for other predictors (i.e., FI, molars, plaque). Additionally, radiographic defect angle and depth may not completely describe the morphology of the defect. Further studies, specifically addressing the tridimensionality of intrabony defect, are needed for a better understanding of its impact on NSPT.

## 5 Conclusions

In conclusion, this study suggests:

- No differences in clinical or patient‐centered outcomes were observed between the 3‐ and 6‐month groups;
- Sites achieving EoT were around 70% in both the 3‐ and 6‐month groups;
- A significant improvement in oral health‐related quality of life is expected after delivery of NSPT;
- Intrabony defects exhibit a reduced healing response following NSPT, with respect to suprabony defect:
- A non‐negligible fraction of intrabony defects may heal with NSPT alone, especially if associated with shallow PDs

## Funding

The authors have nothing to report.

## Conflicts of Interest

The authors declare no conflicts of interest.

## Supporting information


**Data S1:** Bivariate analysis for risk factors for RPs at periodontal re‐evaluation after NSPT.

## Data Availability

The data that support the findings of this study are available on request from the corresponding author. The data are not publicly available due to privacy or ethical restrictions.
