# Supplementary material for: Timing of Re‐Evaluation After Periodontal Therapy: A Randomized Clinical Trial
Source: J Periodontal Res. 2026 Feb 10;61(5):560–71. doi: 10.1111/jre.70086 (PMC13378204; doi:10.1111/jre.70086)
Supplement: Supplementary file 1 — Data S1: Bivariate analysis for risk factors for RPs at periodontal re‐evaluation after NSPT. [file JRE-61-560-s001.docx]

| Factor | OR | 95% CI | p *value* |
| --- | --- | --- | --- |
|  |  |  |  |
| Sex (Male vs Female) | 1.39 | 0.88 – 2.19 | 0.154 |
| Age | 1.02 | 0.99 – 1.05 | 0.073 |
| Smoking (Ref: No Smoker)  Smoking < 10 cigarettes/day  Smoking ≥ 10 cigarettes/day | 1.07  1.13 | 0.57 – 1.98  0.61 – 2.1 | 0.829  0.682 |
| Stage of Periodontitis (4 vs 3) | 1.18 | 0.73 – 1.91 | 0.490 |
| Grade of Periodontitis (C vs B) | 1.54 | 0.95 – 2.52 | 0.078 |
| FMPS Baseline | 1.01 | 0.99 – 1.02 | 0.538 |
| FMBS Baseline | 0.99 | 0.98 – 1.01 | 0.734 |
| Tooth Mobility (Ref: TM=0)  TM 1  TM 2  TM 3 | 0.78  2.35  3.14 | 0.19 – 3.28  0.61 – 9.05  0.84 – 11.8 | 0.743  0.212  0.089 |
| PI Baseline (positive vs negative) | 1.39 | 0.95 – 2.04 | 0.086 |
| BoP Baseline (positive vs negative) | 0.79 | 0.52 – 1.21 | 0.281 |
| PPD Baseline | 2.01 | 1.84 – 2.21 | **<0.0001** |
| Tooth Type (molar vs non molar) | 3.67 | 2.76 – 4.86 | **<0.0001** |
| FI (Yes vs No) | 3.97 | 2.87 – 5.51 | **<0.0001** |
| FMPS Final | 0.98 | 0.97 – 1.01 | 0.251 |
| PI Re-evaluation (positive vs negative) | 1.86 | 1.42 – 2.43 | **<0.0001** |
| Intrabony Defect (Ref: No)  Shallow  Deep | 1.78  5.26 | 1.24 – 2.56  3.23 – 8.57 | **0.002**  **<0.0001** |
| Intrabony Defect Depth (mm) | 1.31 | 1.21 – 1.41 | **<0.0001** |
| Intrabony Defect Angle (Wide vs Narrow) | 1.99 | 0.92 – 4.3 | 0.08 |
| Intrabony Defect Angle (Degrees) | 1.01 | 0.99 – 1.03 | 0.07 |

**Supplementary Material 1 |** Bivariate analysis for risk factors for RPs at periodontal re-evaluation after NSPT. Intrabony defect angle was measured only for intrabony defects and the impact of this variable was analyzed only in infrabony defects.
